# Opinion: hazards faced by macromolecules when confined to thin aqueous films

**DOI:** 10.1007/s41048-016-0026-3

**Published:** 2016-07-22

**Authors:** Robert M. Glaeser, Bong-Gyoon Han

**Affiliations:** Lawrence Berkeley National Laboratory, University of California, Berkeley, CA 94720 USA

**Keywords:** Sample preparation, Cryo-EM, Protein denaturation, Air–water interface

## Abstract

Samples prepared for single-particle electron cryo-microscopy (cryo-EM) necessarily have a very high surface-to-volume ratio during the short period of time between thinning and vitrification. During this time, there is an obvious risk that macromolecules of interest may adsorb to the air–water interface with a preferred orientation, or that they may even become partially or fully unfolded at the interface. In addition, adsorption of macromolecules to an air–water interface may occur even before thinning. This paper addresses the question whether currently used methods of sample preparation might be improved if one could avoid such interfacial interactions. One possible way to do so might be to preemptively form a surfactant monolayer over the air–water interfaces, to serve as a structure-friendly slide and coverslip. An alternative is to immobilize particles of interest by binding them to some type of support film, which—to continue using the analogy—thus serves as a slide. In this case, the goal is not only to prevent the particles of interest from diffusing into contact with the air–water interface but also to increase the number of particles seen in each image. In this direction, it is natural to think of developing various types of affinity grids as structure-friendly alternatives to thin carbon films. Perhaps ironically, if precautions are not taken against adsorption of particles to air–water interfaces, sacrificial monolayers of denatured protein may take the roles of slide, coverslip, or even both.

## Introduction

**Fig. 1:**
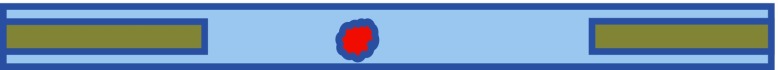
Cartoon representation of an idealized cryo-EM specimen. Note that the particle (*red*), the hole in the carbon film (*brown*), and the thin film of vitreous ice (*blue*) are not drawn to scale. The main point of this cartoon is to illustrate the goal that the particle should be surrounded on all sides by vitrified buffer, and—in particular—the particle should not touch the air–water interface

Under ideal conditions, samples prepared for single-particle electron cryo-microscopy (cryo-EM) can be nearly perfect, in the sense that molecules of interest are surrounded by vitrified buffer, preserved in a life-like state. In effect, such specimens represent an instantaneous snapshot of what the particles were like in solution, a thin slab of which is “removed” and placed onto an EM grid. Figure 1 shows a schematic version of what such an ideal specimen might look like.

The important features of ideal specimens include (1) the slab of vitrified buffer should be no thicker than the depth of field, the value of which depends, of course, upon the resolution that one hopes to obtain (Agard et al. 2014); (2) these very thin samples should be made without there having been an excessive amount of evaporation of water; (3) thinning of the sample should be achieved without much shear stress being applied to the particles; and (4) the macromolecules themselves should not come in direct contact with the air–water interface.

The cartoon in Fig. 1 may be a simplified but nevertheless good representation of what one often obtains when using current methods of preparing grids. Many times, however, the first results are not satisfactory, and further improvement often requires considerable optimization. In those cases, it is important to ask: what might be wrong with “our standard picture”, shown in Fig. 1?

For the present, it will be assumed that evaporation is not a concern, provided that one is able to maintain the grid near to, or below the dew point of the ambient atmosphere until vitrification. In addition, there is not yet experimental evidence that shear forces damage the structures of globular macromolecules, although that remains an issue to keep in mind.

The remaining concern, which is the topic of this paper, is that various things can go wrong with “our standard picture” when proteins of interest come into contact with an air–water interface. To avoid that from happening, there are some ways to keep particles from touching the air–water interface. These include (1) using a surfactant to form a structure-friendly barrier (effectively a cover slip) at the air–water interface, and/or (2) immobilizing the particles onto a support film (effectively a specimen slide) in a structure-friendly way.

## Things that can go wrong at the air–water interface

In all biochemical work, it is a matter of common sense that one should avoid actions that create a large surface-to-volume ratio, for example, vigorous shaking, bubbling, or frothing of the sample. In cryo-EM, it is nevertheless a requirement that one makes very thin films, which have extremely high values of the surface-to-volume ratio. Macromolecules diffusing freely within such thin films, perhaps <100 nm thick, are bound to collide with the air–water interface >1000 times per second (Taylor and Glaeser 2008). As a result, even though the interval between blotting and vitrification may be kept as short as possible, there is plenty of time for something to go wrong. If it does, the actual outcome may not look at all like the picture shown in Fig. 1.

Several examples of what might go wrong have been enumerated previously (Taylor and Glaeser 2008). It is well known, for example, that some types of particles exhibit preferential orientation in cryo-EM images. A likely explanation is that one or more hydrophobic patches on the surface may allow a particle to bind to the air–water interface, in much the same way as it would do to a hydrophobic interaction chromatography bead. Little or no activation energy may be required for this type of binding to occur. Although such binding need not cause a structural change in the particle, it can still make the grid useless for three-dimensional data collection because of preferential orientation of the particles.

More worrisome, of course, is the concern that the particles of interest may become denatured immediately after they adsorb to the air–water interface. The rate at which a denatured-protein layer forms may, in fact, be limited only by the rate of diffusion from the underlying solution to the air–water interface. As background, it was shown by Trurnit (1960)—for the case of serum albumin (diffusion coefficient equal to 6 × 10^−7^ cm^2^/s)—that a few seconds are sufficient for essentially all of the protein molecules within a 10-µm-thick layer of sample to diffuse to, and become completely denatured at the air–water interface. Furthermore, results of a calculation presented in footnote 2 of Trurnit (1960) can be restated to say that a protein solution at a concentration of ~60 µg/mL contains enough material to cover the entire surface of a 10-µm-thick film when the proteins are in a fully extended, unfolded state.

**Fig. 2:**
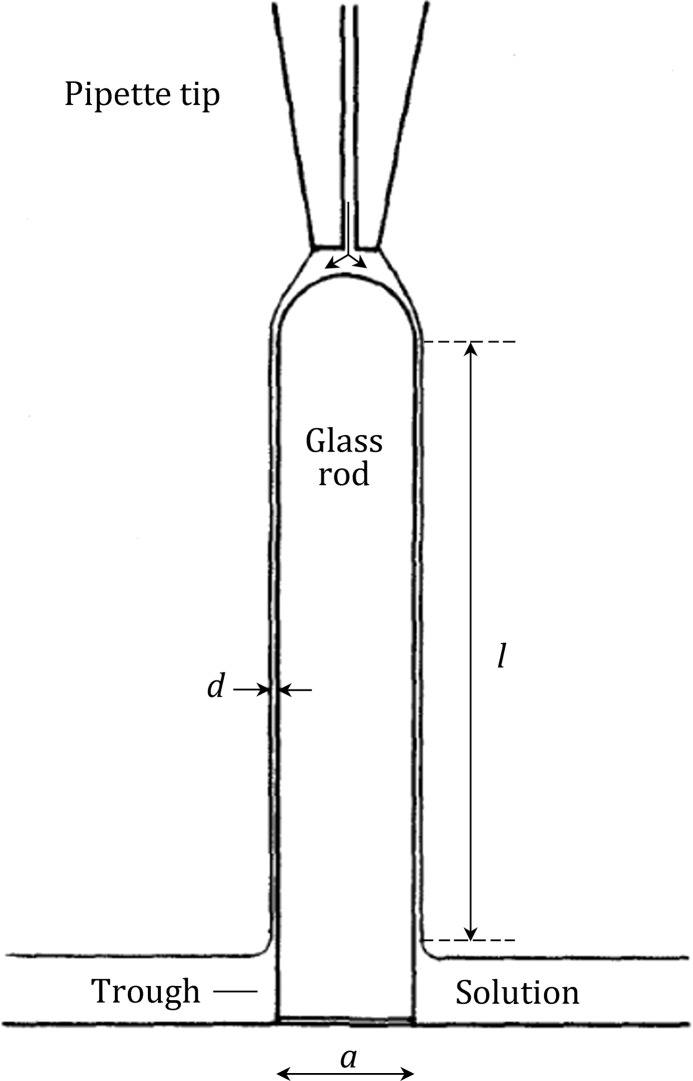
Schematic diagram of the technique introduced by Trurnit (1960) for the quantitative transfer of proteins to the air–water interface of a Langmuir trough. Protein solutions are delivered by pipette to the top of a clean glass rod, of length *l*, after which they flow down as a liquid curtain of thickness *d*, which can be as thin as 10 µm. Protein molecules that diffuse to, and adsorb to, the air–water interface, as the sample flows down the surface of the glass rod, are necessarily delivered to the surface of the trough solution. Reproduced with permission from Trurnit (1960)

Trurnit’s experiments were done by allowing a thin curtain of sample to flow down a glass rod and onto the surface of a Langmuir trough. The setup for doing this is shown schematically in Fig. 2. The rationale is that protein molecules that denature at the air–water interface as the sample flows down the glass rod will remain at the air–water interface when they reach the trough, whereas the rest will mix into the subphase.

Trurnit’s experiment raises the question of why it is possible for protein denaturation to happen so quickly at the air–water interface, when it is such a rare event in bulk solution. The answer must be that the energy landscape for unfolding at the interface is very different than it is in bulk solution.

**Fig. 3:**
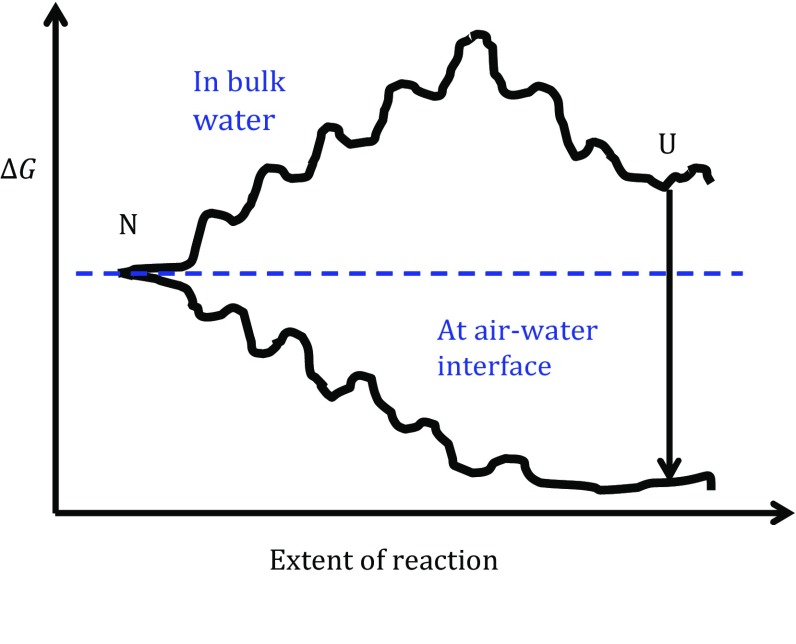
Comparison of schematic free-energy “landscapes” for unfolding of proteins at the air–water interface (*lower curve*) versus when in bulk solution (*upper curve*). The native state, “*N*” is indicated at the beginning of the unfolding reaction, and the unfolded state, “*U*” is indicated at the end of the reaction. The *arrow* on the right-hand side indicates the expectation that the free energy is very favorable for transfer of an unfolded protein from bulk water (*upper curve*) to the air–water interface (*lower curve*). As is argued in the text, this process is nevertheless expected to be rate limited by the low frequency with which unfolded species form in bulk water. As is further argued in the text, however, contact between a protein and the air–water interface can lead to spontaneous unfolding, without any significant activation barriers for completion of the reaction

Proteins in solution are expected to constantly undergo reversible, miniature “sub-globally cooperative unfolding/refolding” events (Maity et al. 2005), such as “fraying” at the ends of helices, or even partial unfolding of small regions at the surface of the protein (Chamberlain et al. 1996). As is indicated schematically by the free-energy diagram in Fig. 3, modeled after Fig. 1 in (Sosnick and Barrick 2011), unfolding is thought to involve a succession of small, destabilizing (uphill) steps, indicated by small bumps (activation barriers) and dips (local minima) in the energy landscape. Only rarely does a protein progress, through a series of such events, to reach a transition state, at which point it can spontaneously proceed downhill into an unfolded state. Although an unfolded protein would be expected to adsorb irreversibly to the air–water interface, that process would be limited by the very slow rate at which denatured species are formed in bulk solution.

Contrary to the situation in bulk solution, denaturation at the air–water interface may involve almost no activation barrier. Rather, as mentioned above, the initial adsorption of a protein (in its native state) may occur spontaneously via a preexisting hydrophobic patch on its surface. Following adsorption to the interface, one can expect local unraveling of “foldons” (Maity et al. 2005) to still occur, just as in bulk solution. Whenever these transient events expose additional hydrophobic groups, they too may bind at the air–water interface. In contrast to the situation in bulk solution, however, each such event is no longer reversible, or at least the cumulative effect is not reversible. As a result, the energy landscape for unfolding native proteins at the air–water interface may be very different from that in bulk solution, as indicated in Fig. 3.

**Fig. 4:**
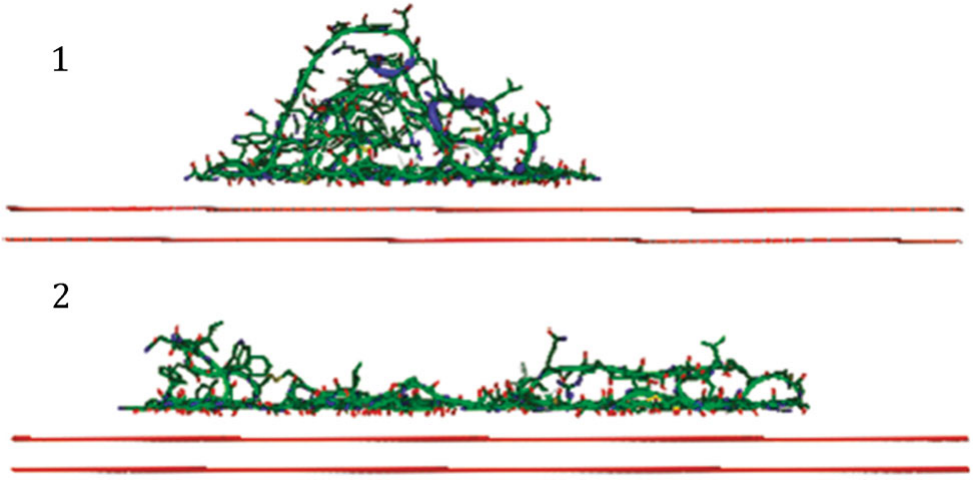
Two examples of unfolded lysozyme structures obtained in molecular dynamics (MD) simulations performed by Raffaini and Ganazzoli (2010). Although the substrate was graphite in these simulations, our opinion is that similar results are to be expected for the air–water interface. It is worth noting that these simulations indicate that an ensemble of unfolded structures is likely to be formed, with the orientation of the protein at the time of initial contact playing an important role in determining the final, unfolded structure. Reproduced with permission

Molecular dynamics simulations of lysozyme on graphite (which can be regarded as a surrogate for air), starting with the protein in its native conformation, suggest that the energy landscape for unfolding is, indeed, monotonically downhill. Furthermore, these simulations suggest that complete unfolding may take only one or a few ns—see Figs. 4A and 6A in Raffaini and Ganazzoli (2010). As shown in Fig. 4, these simulations further suggest that the structure of the unfolded state can vary considerably, depending upon what point on the surface of the lysozyme molecule is the first one to touch the hydrophobic interface.

## Surfactants may form structure-friendly interfaces (slides and coverslips)

It is fairly common practice to add detergents or other surfactants to samples that are used to make cryo-EM grids. This is a requirement when working with solubilized membrane proteins, of course. In addition, it is often something that is done with soluble proteins, especially those that are initially found to be difficult to prepare as cryo-EM specimens.

When the mass of added surfactant is in great excess relative to that of the particle of interest, a monolayer of surfactant will form at the air–water interface before most of the proteins ever get there. Indeed, a surfactant monolayer must already form on the surface of a drop as soon as it emerges from the tip of the pipette. Thus, as was envisioned first for phospholipids (Frederik et al. 1989), other types of surfactant monolayer may also function as a kind of hydrophilic, electron-transparent slide and coverslip.

Surfactant monolayers can be much more structure friendly to proteins than is the air–water interface, and even arguably more structure friendly—as support films—than are glow-discharge treated carbon films. This is because surfactant monolayers are expected to act as a barrier that reduces access to the hydrophobic side of an air–water interface. Nevertheless, proteins may still be able to diffuse into the monolayer and bind via a hydrophobic interaction, unless the (surfactant) surface pressure is unusually high (Quinn and Dawson 1970). At some intermediate surface pressure, unfolding may be prevented even though an initial insertion event still occurs. In this case, it is likely that preferential orientation may again make such specimens of no value for 3-D data collection. On the other hand, the polar groups of most surfactant monolayers are unlikely to interact strongly with soluble macromolecules. Thus, in the event that insertion into the surfactant layer does not occur, the presence of surfactant may offer an opportunity to make the ideal type of specimen imagined in Fig. 1.

## Denatured proteins may also form a structure-friendly layer at the air–water interface

If no surfactant is intentionally added to the sample, it is possible that the protein of interest may itself act as a surfactant. As described above, a denatured-protein monolayer can form within seconds or less, if the sample concentration is tens of micrograms/mL or more. As a result, a monolayer of denatured protein may form on the surface of the sample as it emerges from the tip of a pipette, before it even wets the grid. Furthermore, if formation of such a denatured-protein monolayer is not yet complete at that point, more protein can continue to be added during the time that the sample is incubated on the grid.

Ferritin is an example of a protein that quickly forms such a denatured-protein film at the air–water interface, as was shown by Yoshimura et al. (1994). These authors observed that when a small drop of sample, injected below the surface of a more-dense subphase solution, rises and first touches the air–water interface, it “instantly” forms a denatured-protein layer. Furthermore, molecules in this denatured-protein layer were observed to be so strongly adsorbed to (or entangled with) one another that an intact film could be picked up with a holey-carbon EM grid.

It is instructive to think of such a denatured-protein monolayer as being an electron-transparent slide, in the sense that additional particles could bind to it in their native conformation. As a matter of fact, it is quite commonly said in the literature of protein adsorption at the air–water interface that this is expected to happen—see the third paragraph of Trurnit (1960), for example. In the experiments with ferritin cited above (Yoshimura et al. 1994), the number of native ferritin particles seen on the EM grid increased with the length of time of incubation after the initial, “instantaneous” formation of a denatured-protein layer. In the presence of 10 mmol/L CdSO_4_, even small, 2-D clusters of particles started to form after 3 min, and monolayer crystals were formed after 10 min. Similar results, including the formation of (in this case smaller) 2-D crystal arrays, were also obtained with 20 S proteasome particles from *T. acidophilum*.

## Surfactants may also form structure-friendly coverslips

The potential role of a surfactant-monolayer coverslip is less certain than that of the surfactant “slide” discussed above. What is expected, however, is that such a layer can form as easily on the top surface of a droplet, as it incubates on the EM grid, as it does on the bottom (holey carbon side). What remains unknown, however, is the extent to which the surfactant layer on top of the drop is removed during blotting, and—if removed—the extent to which a new layer forms on the freshly created air–water interface, prior to vitrification.

If, however, there is no coverslip—and no slide, for that matter—during the first second or two after blotting, it may not be possible to get a specimen of the type like that imagined in Fig. 1. The reason, as discussed above, is that many proteins rapidly adsorb to, and even unfold at, a clean air–water interface. Since proteins in solution can be quantitatively removed to the air–water interface within a few seconds from a slab that is ~10 µm thick, the same will happen even faster if the slab is only <100 nm in thickness. Thus, to repeat the point, a surfactant barrier (e.g., cover slip) is required unless the particles of interest have previously been immobilized onto a “slide”.

## The observed number of particles per unit area can be a useful indicator of what is happening

As noted by Taylor and Glaeser (2008), the number of particles per unit area provides a good indication whether everything in the cryo-sample is as expected, i.e., whether things are similar to the ideal case imagined in Fig. 1. If the sample concentration is 1 mg/mL (0.1%), for example, and a slab of thickness equal to ten times the particle size is “taken at random”, then—in projection—the average distance between particles should be equal to ten particle diameters. If the slab is even thinner, then the average distance between particles should be proportionately greater than that. Similarly, if the sample concentration is much lower, perhaps 20 µg/mL, then the average distance between particles should again be proportionately greater.

In some cases, one may see far more particles than there could be if the specimen conformed to the picture shown in Fig. 1. In these cases, one must conclude that one of the following possibilities may be true: (1) Particles were adsorbed to a clean air–water interface, thereby accumulating in large numbers, at the same time suffering little or no structural damage. (2) Particles were adsorbed to a preexisting support film. If a continuous carbon film was not present, however, then the “support film” would have to be some surfactant monolayer, possibly consisting of denatured particles of interest. (3) The sample concentration was increased significantly due to evaporation in the interval between blotting and vitrification. None of these possibilities are necessarily a bad thing, but each of them could be.

In other cases, many particles are seen on the surrounding areas of carbon, but few, if any, in the open holes. In these cases, the suggestion is that intact particles were immobilized by binding to the surrounding carbon film, but not to the air–water interface over a hole. Recall that the expectation for this scenario is that proteins, when diffusing freely within in a thin aqueous film, will be rapidly removed to, and denatured at, a clean air–water interface.

## Other methods for preparing cryo-EM grids can be considered

As mentioned above, the preparation of cryo-EM specimens might be improved by immobilizing particles of interest onto some type of structure-friendly support film (slide). However, the methods currently used to immobilize particles can themselves be criticized. Adsorption to glow-discharge treated carbon films, for example, is used mainly when preparing samples on holey-carbon films has failed. Using evaporated carbon as a “slide” is generally avoided because the carbon film adds noise to the image. In addition, one does not know the mechanism by which particles are adsorbed to carbon films, and, therefore, what the structural consequences might be.

Adsorption to a sacrificial, denatured-protein monolayer is also a mysterious process, at least at the molecular level of understanding. As a result, the mechanism of adsorption and its consequences may be somewhat different for every protein. Finally, as mentioned above, adsorption to a surfactant monolayer probably does not happen in most cases, and when it does, it might result in preferential orientation.

With these shortcomings in mind, a number of different types of “affinity” support films are beginning to be investigated. For example, lipid monolayers that include a Ni–NTA functionality were introduced for affinity binding of his-tagged proteins (Kelly et al. 2008), and a variant of this has been described in which a spacer of poly(ethylene glycol) 2000 (PEG2000) is used to reduce preferred orientation of proteins captured by the lipid monolayer (Benjamin et al. 2016). Furthermore, a “sandwich” strategy has also been described in which his-tagged protein A is first bound to the Ni–NTA lipid monolayer, which in turn can bind antibodies to particles of interest (Kelly et al. 2010). More recently, it has been shown that antibodies can be bound directly to carbon films, preferably after exposing them to a glow-discharge treatment (Yu et al. 2014, 2016). These antibody-based affinity support films are especially attractive, since the use of polyclonal antibodies has promise as a way to avoid preferential orientation.

In another direction, Llaguno et al. (2014) showed that carboxyl groups can be generated on the surface of evaporated carbon films by oxidization with an alkaline solution of potassium permanganate. Primary amine-bearing “affinity ligands” of many kinds can then be coupled to the carboxyl groups.

Yet another approach was taken by Crucifix et al. (2004), Wang et al. (2008), and Wang and Sigworth (2010), who used monolayer crystals of streptavidin as an affinity support film. Monolayer crystals of streptavidin are attractive for many reasons. One appealing feature is that preferential orientation can be avoided simply by randomly biotinylating lysine residues on the surface of the particle of interest (Han et al. 2012). Another desirable feature is that the contribution of the crystalline support film can be subtracted by Fourier filtering the images.

## Even when immobilized on a structure-friendly substrate, particles can still be touched by the air–water interface

**Fig. 5:**
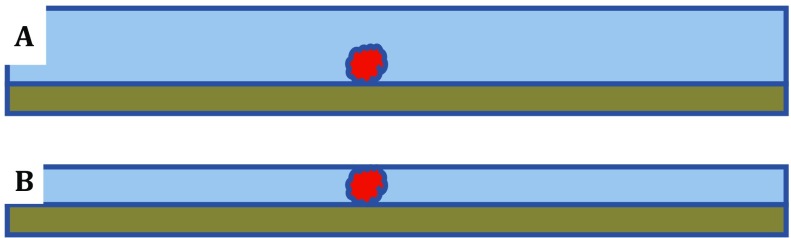
Cartoon to show immobilized particles with the air–water interface well above the particle (**A**) and with the interface touching the particle (**B**). In the ideal case, like that shown schematically in **A**, particles bound to a structure-friendly support film (slide) are not at risk of becoming denatured at the air–water interface as long as the surrounding layer of buffer remains thicker than the size of the particle. As the layer of buffer becomes thinner and thinner, however, the air–water interface must eventually touch the particle, as shown in **B**

In the ideal case, like that shown schematically in Fig. 5A, particles bound to a structure-friendly support film (slide) are not at risk of becoming denatured at the air–water interface as long as the surrounding layer of buffer remains thicker than the size of the particle. As the layer of buffer becomes thinner and thinner, however, the air–water interface must eventually touch the particle, as shown in Fig. 5B. When that happens, the energy landscape for protein unfolding may switch immediately to that, shown in Fig. 3, for a freely diffusing particle that collides with the air–water interface.

Unfortunately, there currently are not known methods to reliably control the sample thickness once it is in the range desired for cryo-EM. This is because van der Waals forces, as pointed out recently by Glaeser et al. (2016), act to further thin an aqueous film once its thickness falls below a certain value, thought to be ~100 nm. If there is no surfactant at the air–water interface, this thinning proceeds until there is complete dewetting at one or more points, accompanied by a corresponding thickening elsewhere.

Random dewetting might be avoided, however, if there is a surfactant at the air–water interface. This is because hydration forces, and possibly other interfacial interactions, produce what is called a “disjoining pressure”—discussed by Glaeser et al. (2016)—that resists further thinning. One might thus speculate that there may be an ultrathin aqueous layer, perhaps only 1 or 2 nm in thickness, between the polar groups of the surfactant and the hydrophilic surface of a particle of interest. The idea is that a surfactant monolayer might act as a thin, flexible coverslip that partially wraps over a particle of interest, still maintaining a very thin hydration layer.

## Summary and conclusions

Classical studies on the formation of denatured-protein monolayers have shown that virtually every protein molecule within a 10-µm-thick, aqueous film is irreversibly transferred to the air–water interface within just a few seconds or less. Work in this field generally expects that additional protein molecules then adsorb (more slowly) to the denatured-protein layer. It is thus noteworthy that one group has even tried to use such denatured-protein monolayers as structure-friendly support films for preparing 2-D crystals for electron microscopy. While one can thus conclude that the initial formation of a continuous, denatured-protein monolayer is not necessarily a bad thing, it seems unlikely that it can serve as a universal method for preparing cryo-EM specimens.

Detergents and other surfactants are, for various reasons, sometimes included along with the macromolecular particle of interest during the preparation of cryo-EM grids. If present at a high enough concentration, these amphiphiles are expected to form a preemptive monolayer at the air–water interface. Such a layer may, at least in some cases, prevent the formation of a denatured-protein monolayer. On the other hand, such layers are not guaranteed to prevent preferential adsorption of particles of interest. Alternatively, they may prevent adsorption of any type.

In view of these problems and concerns, some groups are investigating the use of affinity grids for preparing cryo-EM samples. Affinity binding can produce grids with a large number of particles per unit area, using quite dilute samples. Affinity binding is also expected to be structure friendly, and it can be designed to be virtually free of causing preferential orientation of particles of interest. For affinity grids to be fully effective, however, it may be necessary to find ways to prevent the surrounding aqueous film from becoming so thin that the free air–water interface comes into contact with the immobilized particles prior to vitrification.
